# Debridement, antibiotics, and implant retention in non-oncological femoral megaprosthesis infections: minimum 5 year follow-up

**DOI:** 10.1186/s40634-022-00469-9

**Published:** 2022-04-11

**Authors:** A. Asokan, M. S. Ibrahim, J. W. Thompson, F. S. Haddad

**Affiliations:** Department of Trauma and Orthopaedic Surgery, University College Hospital, 235 Euston Road, Fitzrovia, London, NW1 2BU UK

**Keywords:** Megaprosthesis, Periprosthetic infection, DAIR, Debridement, Antibiotics, Implant retention

## Abstract

**Purpose:**

Megaprostheses are increasingly utilised outside of the oncological setting, and remain at significant risk of periprosthetic joint infection (PJI). Debridement, antibiotic, and implant retention (DAIR) is an established treatment for PJI, however its use in non-oncological patients with femoral megaprostheses has not been widely reported. There are significant differences in patient physiology, treatment goals, and associated risks between these patient cohorts.

**Methods:**

We identified 14 patients who underwent DAIR for a PJI of their femoral megaprostheses, between 2000 and 2014, whom had their index procedure secondary to non-oncological indications. Patients were managed as part of a multidisciplinary team, with our standardised surgical technique including exchange of all mobile parts, and subsequent antibiotic therapy for a minimum of 3 months. Patients were followed up for a minimum of 5 years.

**Results:**

Patients included six proximal femoral replacements, five distal femoral replacements, and three total femoral replacements. No patients were lost to follow-up. There were six males and eight females, with a mean age of 67.2 years, and mean ASA of 2.3.

Nine patients (64.3%) successfully cleared their infection following DAIR at a minimum of 5 year follow-up. Five patients (35.7%) required further revision surgery, with four patients cleared of infection. No patients who underwent DAIR alone suffered complications as a result of the procedure.

**Conclusions:**

The use of DAIR in these complex patients can lead to successful outcomes, but the risk of further revision remains high. The success rate (64.3%) remains on par with other studies evaluating DAIR in megaprostheses and in primary arthroplasty. This study indicates judicious use of DAIR can be an appropriate part of the treatment algorithm.

**Level of evidence:**

II

## Introduction

Megaprostheses are increasingly utilised to manage significant bone loss secondary to trauma, revision arthroplasty, and malignancy [1, 2]. Their use in the lower extremity has transformed the functional prognosis of a patient through limb salvage and immediate postoperative weightbearing, where previously they may have undergone amputation for the same pathology. Megaprostheses are established implants in oncological settings, whereas their use in trauma and revision arthroplasty is growing [3–5]. Relative to the hip and knee, megaprostheses would include proximal-, distal-, and total femoral replacements, in addition to proximal tibial replacements. However, in this manuscript, discussion is limited to femoral implants.

Periprosthetic joint infection (PJI) has a low incidence of 1–2% in conventional arthroplasty, but remains a potentially devastating complication with implications on both patient and surgeon [6–8] Comparatively, infection rates are significantly higher in lower extremity megaprostheses, with incidences between 3 and 19.5% [9–14], with some studies reporting as high as 43% in previously infected megaprostheses [15]. Treatment strategies to combat PJI range from surgical debridement and irrigation with implant retention (DAIR – Debridement, Antibiotics, and Implant Retention), to single-stage or two-stage revision, to amputation [2, 16–20].

Two-stage revision is the benchmark treatment strategy for PJI in conventional arthroplasty, but comes with significant physiological demands, morbidity, and financial costs. In megaprostheses, the complex nature of massive bone loss, deadspace, and soft tissue cover, create an exponentially greater challenge, for which two-stage revision may be too great an undertaking both technically for the surgeon and physiologically for the patient. Alternatively, DAIR is an accepted treatment for acute infections, and often first-line management in PJI, but its use in megaprostheses has not yet widely been explored.

The relatively low prevalence of megaprostheses leads to a paucity of data surrounding treatment strategies and their outcomes for PJI. A number of studies have evaluated these outcomes in oncological patients [1, 2, 10, 21–24], however there is limited data in non-oncological cases. There is significant heterogeneity between these two cohorts, which must be considered separately. Oncological patients commonly require extensive soft-tissue resections, which is a key contributor to increased infection risk. Additionally, they may also receive adjuvant chemo-radiotherapy, and have extended operating times. These factors have been suggested in a number of studies to contribute to the higher PJI rates seen in oncological patients compared with revision arthroplasty [14, 25, 26].

To the authors’ knowledge, there is only one other study which evaluates outcomes of PJI in non-oncological megaprostheses, however does not differentiate which patients underwent DAIR, single- or two-stage revision, or amputation [1].

Therefore, this study aims to report the outcomes for patients undergoing DAIR for PJI in megaprostheses which were performed for non-oncological indications, and describe our technique.

## Methods

### Patients

Prospective data was collected on any patients with a proximal, total, or distal femoral megaprosthesis (Global Modular Replacement System; Stryker, Kalamazoo, MI, USA), who underwent a DAIR procedure for PJI between 2000 and 2014. Only patients who underwent their index megaprosthesis procedure for non-oncological indications were included. Index procedures were performed at multiple centres, however, all DAIR procedures were carried out at our institution, which is a tertiary referral centre for PJI and complex lower-limb arthroplasty. Patients were identified to have infection if they met clinical criteria including pain, fevers, elevated inflammatory markers, and positive cultures [27]. Any patients who had previous infection-related surgery such as single-stage or two-stage revisions prior to DAIR were excluded. All patients were followed up for a minimum of 5 years.

### Outcome measures

We defined successful treatment as patients who remained clear of infection at 5 year follow-up without the need for additional increasing surgical intervention i.e. proceeding to single or two-stage revision. Any patients who required revision surgery or died as a result of PJI were deemed to have failed DAIR treatment. Additional intra- or post-operative complications were noted such as dislocation or venous thromboembolism, but they were not deemed failure of treatment.

### Treatment strategy

All patients suspected to have PJI, were appropriately assessed and blood samples were obtained including blood cultures, Full Blood Count (FBC), C-reactive protein (CRP), and Erythrocyte Sedimentation Rate (ESR). Relevant radiographs were acquired, and if indicated, further imaging with bone scan or single-photon emission computer tomography (SPECT) scan were performed to look for loosening. All suspected patients were managed in a multidisciplinary team (MDT) including senior surgeons, microbiologists, infectious disease experts, radiologists, and therapists. MDT consensus was sought prior to any interventional step [17, 28].

DAIR followed our standardised surgical technique. Five or more intra-operative tissue samples were collected prior to any antibiotics, and were sent for extended culture, sensitivities, and Gram staining to guide antibiotic therapy. Any membrane, if present, was collected from the acetabular, femoral and tibial sites. Extensive debridement of diseased tissues was performed including excision of any sinus. Any mobile parts were discarded, and remaining implants irrigated with copious betadine, hydrogen peroxide, and high-volume lavage with a minimum of six litres of normal saline. Following which, new instruments were used, the site rewashed with lavage, and new mobile parts were exchanged. In cases of total femoral replacements, all femoral components were exchanged, but acetabular and tibial components were not. All cases utilised Stimulan (Biocomposites, Keele, UK) as antibiotic beads and/or void filler.

Post-operatively, patients were commenced on intravenous antibiotics (Teicoplanin) as per local protocol, and tailored once intra-operative cultures were available. Antibiotic choice and length of duration was decided by microbiologists in the MDT, guided by culture sensitivities. Antibiotics were converted to oral at 2 weeks, and continued for a minimum of 3 months, up to 6 months. Serial CRP and ESR measurements were taken whilst on antibiotic therapy to assist clinical evaluation of effectiveness. Rising inflammatory markers, increased pain, or a discharging wound were indications for further procedure. This again involved MDT discussion, and the decision for further debridement or progression to revision was made.

All procedures and outcomes were recorded as part of routine patient care, for which written informed consent was collected from all patients prior to their procedures. The study was prospectively reviewed by the hospital review board who advised further Research Ethics Committee approval was not required.

## Results

### Patient demographics

**Fig. 1:**
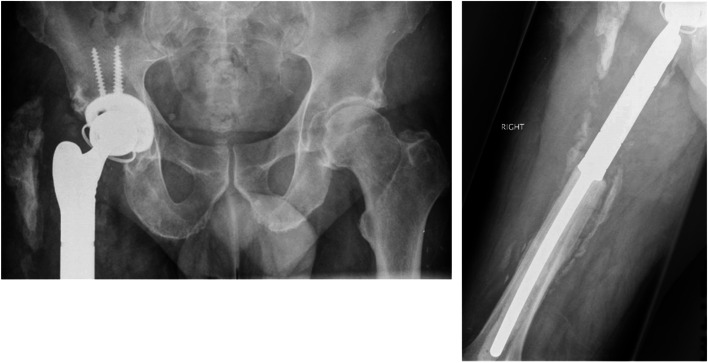
Radiographs of a patient with a Proximal Femoral Replacement taken prior to DAIR surgery

**Fig. 2:**
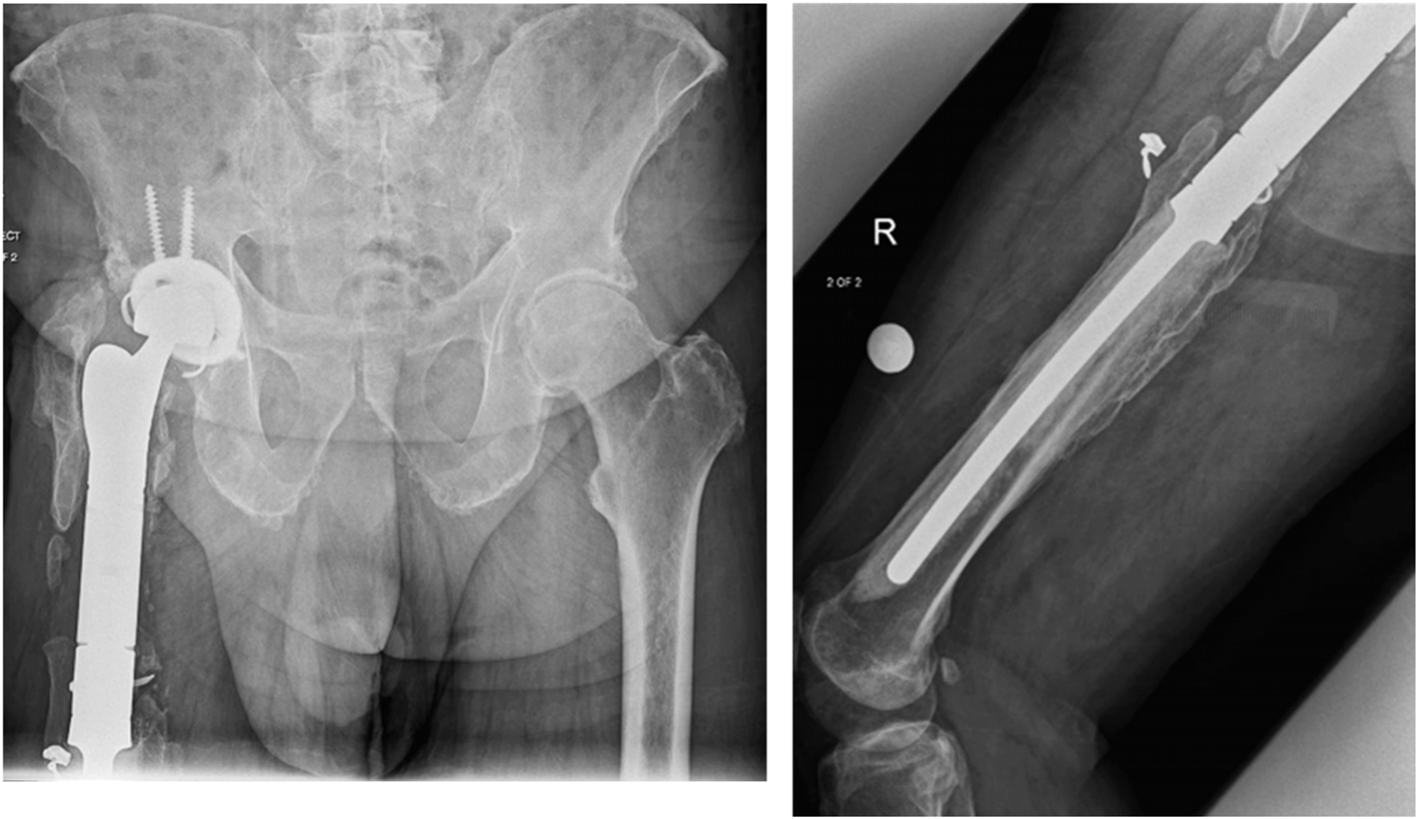
Corresponding radiographs taken at 132 month follow-up, post-DAIR surgery

A total of 14 patients were evaluated in this study, represented in Table 1, including six proximal femoral replacements, five distal femoral replacements, and three total femoral replacements. This included six males and eight females, with a mean age of 67.2 years (range 47–82), and mean American Society of Anaesthesiologists (ASA) grading of 2.3 (range 1–3). Mean follow-up was 93 months (range 64–136 months), with no patients lost to follow-up. Radiographs of a typical PFR included in this study are shown in Figs. 1 and 2.

**Table 1:** Patient Demographics, diagnosis, management and outcomes

Patient	Age/sex	Prosthesis	Indication	Time to infection (days)	Microorganism(s)	Antibiotic Choice	N^o^ of debridement	ASA	Outcome	Complications	Follow Up (months)
1	57 F	PFR	Osteolysis	12	Staph A Pseudomonas	Rifampicin & Ciprofloxacin	2	2	Clear		136
2	63 M	PFR	Fracture	34	CNS	Vancomycin & Gentamicin	1	2	Clear		98
3	82 F	PFR	Fracture	118	Enterococcus	Amoxicillin	1	3	Clear		70
4	47 M	PFR	Infection	38	Staph A Pseudomonas	Rifampicin & Ciprofloxacin	2	2	2-Stage - Clear	DVT & PE	84
5	59 M	PFR	Osteolysis	16	CNS	Linezolid	1	2	Clear		128
6	67 F	PFR	Infection	90	Staph A Strep	Flucloxacillin & Amoxicillin	1	3	2-Stage - Clear		99
7	69 F	DFR	Fracture	21	MRSA	Linezolid	1	2	Clear		64
8	74 F	DFR	Fracture	43	MRSA	Linezolid	1	3	2-Stage - Clear	Antibiotic Intolerance	114
9	59 M	DFR	Aseptic Loosening	68	Staph A Enterococcus	Amoxicillin & Flucloxacillin, Followed by Doxycycline	2	1	Clear		130
10	79 F	DFR	Aseptic Loosening	145	CNS	Rifampicin & Ciprofloxacin	1	3	Clear		66
11	76 M	DFR	Fracture	109	Staph A Enterococcus	Amoxicillin & Flucloxacillin	1	3	2-Stage - Clear		81
12	72 F	TFR	Infection	78	CNS Pseudomonas Strep	Rifampicin & Ciprofloxacin	3	2	1-Stage & ABx Suppression	Dislocation	Died 4 Years Later
13	68 M	TFR	Fracture	14	Staph A	Rifampicin & Ciprofloxacin	1	2	Clear		112
14	70 F	TFR	Aseptic Loosening & Fracture	37	CNS MRSA	Linezolid	1	2	Clear		129

*PFR* Proximal Femoral Replacement, *DFR* Distal Femoral Replacement, *TFR* Total Femoral Replacement, *Staph A Staphylococcus Aureus*, *CNS* Coagulase Negative Staphylococcus, *MRSA* Methicillin Resistant *Staphylococcus Aureus*, *Strep* Streptococcus, *Abx* Antibiotics, *DVT* Deep Vein Thrombosis, *PE* Pulmonary Embolism

The indications for primary procedure are represented in Table 1 with fracture being the most common cause (*n* = 6, 42.9%). The mean age of prosthesis at presentation of infection was 58.8 days (range 12–145). All patients presented with clinical signs of PJI, and pain was the more prevalent symptom. Wound complications including purulent discharge from the surgical site was more commonly seen in early infections. On investigation, both CRP and Neutrophil counts were elevated in all patients. The microbial differential found in these patients is represented in Table 2, with the majority being monomicrobial infections (79%), whereas three (21%) patients grew polymicrobial infections. *Staphylococcus Aureus* was found to be the most prevalent organism with six cases (42.9%) of infection. No fungal infections were found, and no patients were culture negative.

**Table 2:** Microbial differential and prevalence. *N* ≥ 100% due to polymicrobial infections

Microbe	N
*Staphylococcus Aureus*	6 (42.9%)
Coagulase-Negative Staphylococcus	5 (35.7%)
Methicillin Resistant *Staphylococcus Aureus*	3 (21.4%)
Pseudomonas	3 (21.4%)
Beta-Haemolytic Streptococcus	3 (21.4%)
Enterobacter	3 (21.4%)

All patients underwent a DAIR procedure as first-line treatment for PJI. No patients received parenteral antibiotics pre-operatively, and were treated with a culture-driven antibiotic regimen, with the combination of Rifampicin and Ciprofloxacin being the most commonly used (*n* = 5, 35.7%).

### Patient treatment outcomes

A total of nine patients (64.3%, *n* = 14) have survived beyond 5 years without the need for further surgery, which is defined as successful treatment in this study. Two patients in the successfully treated group required two debridements, but eventually were clear of infection. Across all patients, four required more than one debridement (mean 1.4, range 1–3). No complications were noted in this group.

Five patients (35.7%) required revision surgery, defined as unsuccessful treatment in this study. Four patients underwent two-stage revision and were then clear of infection at five-year follow-up. One patient underwent single-stage revision but required long-term antibiotic suppression. Therefore of the 14 patients in total, 13 were clear of infection at 5 year follow-up. Within this unsuccessful group, three complications were noted (Table 1). These included one venous thromboembolism which received appropriate medical therapy; one patient developed antibiotic intolerance part-way through their treatment which required an adjustment of their antibiotics; and one patient suffered a hip dislocation which underwent closed reduction, however the patient died 4 years later. None of these complications were felt to be related to their original DAIR procedure.

In the same study period, 27 patients were not suitable for DAIR and proceeded directly to revision surgery due to a number of reasons including soft-tissue complications which require additional soft-tissue procedures, and/or patient preference. Two patients were managed non-operatively with antibiotic suppression alone due to frailty.

## Discussion

Our study reports a 64.3% success rate of DAIR used in non-oncological femoral megaprostheses with a minimum of five-year follow-up. Megaprostheses have traditionally been utilised within oncological orthopaedics, but their use has expanded to traumatic bone loss and revision arthroplasty. Associated PJI in these implants remains a complex pathology, without an established consensus on treatment strategy, in part due to the rarity of the implants.

There is limited literature surrounding the outcomes of DAIR in megaprostheses outside of the oncological setting, with only one other study reporting on non-oncological megaprostheses. Ercolano et al. performed a retrospective analysis of 31 patients with PJI of a megaprosthesis, with 15 undergoing DAIR, and the rest either single- or two-stage revision, or amputation [1]. The index procedures were carried out in 12 oncological cases, and 19 non-oncological indications, but they do not differentiate between which of these cohorts underwent which surgical treatment method. Nevertheless, they report a success rate of 40% with DAIR alone, and report greater success in patients who underwent multiple debridements as opposed to single. In contrast, our study reports a 64.3% success rate, with most patients requiring only one debridement, at a significantly longer follow-up time (mean 93 months vs 39.6 months). It should be noted although not specific to their DAIR cohort, Ercolano et al’s study includes oncological patients (38.7%), and chronic (> 6 months) infections (48.4%); both of which are detrimental factors, and may contribute to their increased failure rate.

In the oncological setting, there are a greater number of studies evaluating DAIR, though there remains significant variation on success rates between 6% to 75%. The largest series of PJI in oncological megaprostheses was reported by Jeys et al. [10] and includes 68 patients who underwent DAIR, with only a 6% success rate; whereas Peel et al. [24] report a 75% success rate in their series of eight patients. More comparably, both Allison et al. [21] and Dhanoa et al. [22] report a 42% success rate in their series of 15 and seven patients, respectively. Most recently, Nucci et al. [2] performed a pooled analysis of 53 patients (both oncological and non-oncological) and found a 44.9% success rate, in comparison to 52.9% in single-stage revision and 72.3% in two-stage. These studies highlight the heterogeneity in reporting, but allows some benchmarking for our study, which shows a greater success rate. Oncological patients provide additional challenges including perioperative chemo-radiotherapy, difficult soft-tissue cover, wide resection margins, and poor physiological conditions; all of which have potential to increase failure rates and are confounding factors. At present, two-stage revision remains the benchmark in oncological patients.

In acute PJI of primary hip and knee arthroplasty, DAIR is often utilised as first-line treatment, though its efficacy remains debated [16]. Success rates between 26% to 98% have been reported in the literature, with a systematic review of 710 patients reporting a 46% success rate [16, 29, 30]. It would be reasonable to expect lower success in megaprostheses over primary arthroplasty given the aforementioned risk factors, however, our reported success rate of 64.3% remains comparable to many studies which evaluate DAIR in primary arthroplasty. A number of treatment principles from PJI in primary arthroplasty are applied to megaprostheses. Firstly, utilising the multidisciplinary team to discuss and decide management decisions, with subsequent standardised treatment is a strength of this study, and noted to be of benefit in other studies [28]. Secondly, a routine part of the surgical technique used in this study includes the exchange of any modular components, which has been highlighted as an important factor for improving eradication in other studies [16, 31].

It is well recognised that a key component to managing PJI is targeted antibiotic therapy, ideally based upon intra-operative tissue samples [32, 33]. The issue of culture-negative PJI poses significant challenges [34]. In our study, empirical antibiotics were started only once intra-operative samples were obtained, then adjusted based upon culture sensitivities, in keeping with recognised strategies at the time [35]. However, the use of peri-operative antibiotics in PJI has been contentious, and guidance has since changed. Recent evidence has suggested antibiotic administration on induction does not adversely affect culture yields to a significant degree, and remains an important defence against surgical site infections [36, 37]. In 2018 the International Consensus Meeting recommended that peri-operative antibiotics in revision arthroplasty should not be routinely withheld, and should be guided by clinical suspicion for PJI [38]. These recommendations were made after our study period. In addition, with expanded use of novel technologies such as molecular testing, the overall accuracy of microbial diagnosis is greatly improved, irrespective of peri-operative antibiotic use, which may prove an important adjunct in the management of PJI [32, 39, 40].

DAIR is considered to work best in more acute infection, but again heterogeneity of reporting within the literature makes this assessment difficult [41]. In our study, all five patients who failed DAIR treatment presented with infections greater than 4 weeks from primary surgery (mean 71.6 days, range 38–109). Whereas, in the successful group, five patients presented at greater than 4 weeks, and four patients within 4 weeks; with an overall mean of 51.7 days (range 12–145). Although no statistical analysis has been performed, this would suggest DAIR does indeed work better in patients who present more acutely, however there may be a role in delayed presentations.

No patients suffered complications as a direct result of undergoing DAIR, however five patients went on to escalated procedures. Four of these cleared their infection following two-stage revision, and remained clinically well at a minimum of 5 year follow-up. One patient underwent single-stage revision, with subsequent antibiotic suppression therapy life-long, but died 4 years later. Three patients suffered other complications including DVT/PE (*n* = 1), dislocation (n = 1), and antibiotic intolerance (n = 1); all of which were patients in whom further revision surgery was required. No patients had a delay or detrimental outcome from treatment with DAIR.

*Staphylococcus Aureus* was the most prevalent organism in this cohort, found in six (42.9%) patients. MRSA is cited as a cause for poor prognosis, leading to greater debridements and failure [31, 42–44]. In our cohort, three (21.4%) patients were found to have MRSA, of which two cleared their infection following a single debridement, and one required two-stage revision to clear their infection. It is encouraging to see successful outcomes with DAIR in this difficult pathogen cohort.

There are several limitations to be acknowledged within this study. Firstly, the inherent rarity of these implants leads to a limited sample size, which does not allow for statistical analysis, but given the rarity – we consider these results clinically significant. Secondly, these were performed at a single centre, by a single surgeon (FSH), however this was at a tertiary centre for PJI and managed via an MDT approach. More robust results would be gained from a multicentre study. Thirdly, patients were not standardised past indication for primary procedure and undergoing DAIR, thus there may be other confounding factors influencing their outcome. Fourthly, the very nature of undergoing DAIR instead of a revision procedure may be indicative of a patients’ poor physiological status, which in itself could be a confounding factor. Finally, additional information on quality-of-life following DAIR in the form of quality-of-life surveys or other scoring systems was not sought, and may provide an additional metric to guide the treatment algorithm.

## Conclusion

In conclusion, this study has shown a 64.3% success rate using DAIR surgery to treat PJI in patients with non-oncological femoral megaprostheses. This is comparable to other studies that evaluate PJI in both megaprostheses and primary arthroplasty. It is recommended a patient specific, multi-disciplinary approach, with targeted anti-microbial therapy and thorough tissue debridement is used. This study adds to the limited literature in the rare demographic.
